# Safety and Efficacy of Anidulafungin for Fungal Infection in Patients With Liver Dysfunction or Multiorgan Failure

**DOI:** 10.1093/ofid/ofw241

**Published:** 2016-11-12

**Authors:** Anita Verma, Georg Auzinger, Michal Kantecki, James Campling, Dean Spurden, Fran Percival, Nigel Heaton

**Affiliations:** 1 Department of Medical Microbiology; 2 Institute of Liver Studies, King’s College Hospital, Denmark Hill, London, United Kingdom; 3 Pfizer PFE, Paris, France; 4 Pfizer Limited, Tadworth, Surrey, United Kingdom; 5 pH Associates, Marlow, United Kingdom

**Keywords:** anidulafungin, antifungal agents, echinocandins, invasive candidiasis, liver diseases

## Abstract

**Background:**

The objective of this study was to review our clinical experience on the safety and efficacy of anidulafungin, an echinocandin antifungal, in the treatment of invasive fungal infections (IFIs) in patients with moderate to severe abnormal liver function tests or multiorgan failure and IFI, in a large United Kingdom Liver Centre.

**Methods:**

The clinical records of the first 50 consecutive patients treated for IFI with anidulafungin between January 7, 2009 and March 2, 2011 were analyzed. Data were collected on demographics, underlying disease, disease characteristics, hematological and biochemical parameters, IFI, concomitant bacterial and viral infections, response to anidulafungin, and anidulafungin-related adverse events.

**Results:**

The patients’ median age was 54.3 years (range, 19.6–75.9); 60% were male. Twenty-two (44%) patients were liver transplant recipients. Others had hepatopancreaticobiliary disease (n = 15, 30%) or chronic liver disease (n = 11, 22%). Invasive fungal infection (predominantly *Candida* spp) was proven in 36 (72%) patients, probable in 14 (28%). Of 46 evaluable patients, 35 (76%) had a favorable anidulafungin treatment outcome. Forty-nine (98%) had abnormal liver function tests (LFTs) pretreatment; 31 (62%) had ≥1 LFT raised to ≥2× baseline during anidulafungin treatment.

**Conclusions:**

In this highly specialized group of patients, anidulafungin treatment was efficacious and well tolerated by those with decompensated liver disease, multiorgan failure, and high-risk liver transplant with proven or probable IFI.

Anidulafungin is an echinocandin antifungal agent that is indicated for the treatment of invasive candidiasis, frequently a life-threatening disease [1]. Clinical trials have demonstrated the efficacy and safety of anidulafungin for the treatment of invasive candidiasis [2, 3] in critically ill patients [4]. Earlier case series have reported on anidulafungin use in the critically ill, in 21 hematology patients with concomitant hepatic or renal impairment [5], and in a larger US cohort, 25 of the patients treated empirically had hepatic dysfunction. To the best of our knowledge, real-world outcomes in a select cohort of patients with primary liver disease, moderate to severe liver dysfunction, and after liver transplant have thus far not been reported [6, 7].

Patients with severe liver disease and multiorgan failure are at high risk of invasive fungal infections (IFIs). Unlike other echinocandins [8, 9], anidulafungin is not metabolized by the liver but undergoes slow nonenzymatic degradation in the blood instead [6], and so no dose adjustments are required or recommended for patients with any degree of hepatic dysfunction [7, 10, 11]. Furthermore, unlike caspofungin [8] and micafungin [9], anidulafungin has been shown to have no clinically important drug interactions [10]; in particular, unlike caspofungin [8], no dose adjustments or additional monitoring are required when it is used concomitantly with ciclosporin [12] and tacrolimus [13]—calcineurin inhibitor drugs commonly used as first-line immunosuppressant therapy in liver transplant patients.

Kings College Hospital NHS Foundation Trust is a large tertiary referral institution with a comprehensive service covering liver transplantation, general hepatology, and hepatopancreaticobiliary surgery. It has a particular reputation for setting standards in the treatment of acute liver failure with a dedicated 19-bed Liver Intensive Care Unit and an additional 70 ward-level beds. More than 250 adult and pediatric transplants are performed annually [14]. Common conditions that require liver intensive care include acute liver failure, hepatobiliary surgery, acute or chronic liver disease, necrotising pancreatitis, and postliver transplant care. In July 2009, anidulafungin was introduced onto the hospital’s formulary, and from September 2009 it was used as the first choice antifungal for the treatment of invasive candidiasis in patients with liver disease and multiorgan failure. Safety and efficacy were evaluated in the first 50 patients treated with anidulafungin. This is the first report of the “real world” clinical effectiveness of anidulafungin in a cohort of patients with liver disease and multiorgan failure, and to the best of our knowledge it is the largest cohort of patients with liver disease reported to date.

## PATIENTS AND METHODS

This study was conducted by retrospective, observational review of hospital medical and electronic records. Consecutive patients who had been admitted to the Liver Unit and had been prescribed anidulafungin for IFI between July 1, 2009 and February 3, 2011 were included in the study. Patients who had participated in any other interventional clinical trial during the episode of sepsis when anidulafungin was used and patients who had received anidulafungin prophylactically or empirically were excluded. Patient consent was not sought for this study as permitted by United Kingdom Data Protection legislation [15] because it involved no intervention, and the data were collected by NHS staff with access to the patient case notes as part of normal clinical care. The study was given a favorable opinion by the Central London Research Ethics Committee (reference 10/H0716/65) and approved by the King’s College Hospital NHS Trust Research and Development committee.

All study data were sourced retrospectively between February and May 2011 from patients’ case notes and electronic databases at King’s College Hospital. The data collected included patient and disease characteristics, Model for End-Stage Liver Disease (MELD) score [16] for patients with chronic liver disease, biochemical parameters at start date to 5 days after stopping anidulafungin; liver function tests (LFTs), international normalized ratio, platelets, white blood count, C-reactive protein, creatinine, urea, concomitant viral and bacterial infections, comorbidities, concomitant drugs, details of invasive fungal infection site and infecting species, previous antifungal exposure within 30 days before start of anidulafungin, outcome of antifungal treatment, clinical and microbiological response to anidulafungin treatment, anidulafungin-related adverse events, and hospital mortality.

*Candida* isolates in blood culture and in sterile body fluids were identified to species level, but isolates from sputum, bronchoalveolar lavage, throat swabs, and wound swabs were not identified to species. “Proven invasive candidiasis” was defined as isolation of *Candida* species (spp) from peripheral blood or normally sterile body site or histopathological or culture evidence of tissue invasion. “Probable invasive fungal infection” was based on inclusion of a host factor that identified patients at risk, clinical signs and symptoms consistent with the disease entity, mycological evidence that encompassed culture and microscopic analysis, and also indirect tests, such as antigen detection.

Study data were recorded on study case report forms. The anonymized-coded case report forms were then transcribed into the study database and analyzed using descriptive statistics. Both distributions and descriptive statistics of both central tendency and dispersion are presented for quantitative and ordinal variables, whereas nominal variables are described with frequencies and percentages.

The primary outcome measure was the proportion of patients with a “favorable outcome” of anidulafungin treatment. Secondary outcome measures included the proportion of patients with a favorable clinical response, unfavorable outcome, lack of clinical response, death (all causes), death attributable to fungal infection, and death cause unrelated to fungal infection. To minimize bias from clinical judgment, the main study outcomes were defined as follows: (1) favorable outcome was defined as “favorable clinical response” and “documented or presumed microbiologic eradication”; (2) favorable clinical response was defined as the clinical resolution of signs and symptoms of infection or the transition to oral antifungal agent for completion of therapy and with no need to change or add to parenteral antifungal therapy; (3) documented or presumed microbiologic eradication was defined as 2 negative follow-up blood cultures for bloodstream infections or a successful clinical response without follow-up cultures for other infections; (4) “unfavorable outcome” was defined as the need to change to another antifungal agent because of a lack of clinical response or death due to the fungal infection or microbiologic persistence of the fungus or superinfection with a new *Candida*, *Aspergillus*, or other fungal strain occurring after at least 3 days and up to 14 days of anidulafungin therapy, or a lack of follow-up data about clinical and microbiologic responses at the end of anidulafungin therapy; (5) “abnormal” LFT results were defined as those above the normal range (bilirubin >20 μmol/L, aspartate transaminase >50 IU/L, alkaline phosphatase >130 IU/L, and gamma glutamyl transferase >55 IU/L); (6) deterioration in liver function was defined as at least a 2-fold increase in 1 or more LFTs during anidulafungin treatment compared with the start of treatment.

For patients who died, deaths were classified as attributable to fungal infection or causally related to anidulafungin based on clinical opinion, consistent with requirements of the European Medicines Agency Guideline on Good Pharmacovigilance Practices module VI [17].

## RESULTS

### Patient Characteristics

The median age of the 50 study patients was 54.3 years (range, 19.6–75.9), and 60% were male. The majority of enrolled patients were liver transplant recipients (n = 22, 44%, 3 of these had acute liver failure), patients with hepatopancreaticobiliary disease (n = 15, 11 of these had malignancy), chronic liver disease (n = 11), acute liver failure (n = 1), and liver trauma (n = 1). Of the 30 patients with a MELD [16] score, 13 (43%) presented with decompensated liver disease (ie, a MELD score ≥30). Median length of hospital stay was 69 (interquartile range [IQR], 50–99) days. Median length of Liver Intensive Care Unit stay was 20 (IQR, 7–38) days during their admission.

Most of these patients had comorbidities and complex disease presentations. All patients presented with at least 1 marker of illness severity, and 27 (54%) patients presented with 5 markers or more. The main predisposing factors defining illness severity were major surgery (n = 37, 74%) and renal failure (n = 31, 62%) (Table 1). All 50 patients (100%) met the criteria for systemic inflammatory response syndrome (SIRS) [18]. One or more relevant comorbidities were present in 48 patients (96%), with history of alcohol abuse (n = 19, 38%), diabetes (n = 18, 36%), and malignancy (n = 12, 24%) being the most prevalent (Table 2). Thirty-nine patients (78%) had 2 or more comorbidities. Forty-six percent of the cohort had a viral infection, 9 (39%) of which tested positive for cytomegalovirus. More than 40% of patients were colonized with *Candida* (16% *Candida albicans*, 10% *Candida glabrata*, 16% other *Candida* spp). Seventy-six percent of patients (n = 38) were on antifungal prophylaxis (fluconazole in 35 [70%] patients) within 30 days before start of anidulafungin.

**Table 1.** Markers of Illness Severity Before Starting Anidulafungin

Markers of Illness Severity* (n = 50)	Number of Patients	Percent
Major surgery	37	74%
Renal failure	31	62%
Total parenteral nutrition	26	52%
Central line on admission	25	50%
Concomitant viral infection	23	46%
Upper gastrointestinal bleed	21	42%
Dialysis	20	40%
Intubated on admission	15	30%

*Not mutually exclusive.

**Table 2.** Presence of Relevant Comorbidities

Comorbidities* (n = 50)	Number of Patients	Percent
History of alcohol abuse	19	38%
Diabetes	18	36%
Malignancy	12	24%
Hepatitis C	5	10%
Previous liver transplant	9	18%
Renal failure	6	12%
Multiorgan failure	6	12%
Liver abscess	4	8%
Hepatic artery thrombosis	3	6%
Other	26	52%

*Not mutually exclusive.

There were 36 (72%) proven cases of IFI and 14 (28%) probable cases. The most common site of infection was intra-abdominal in 24 (48%) patients and blood in 11 (22%) patients (sites of infection were not mutually exclusive). Most of the infections were due to *Candida* spp, predominantly *C albicans* (n = 17, 34%) and *C glabrata* (n = 17, 34%); other species were *Candida parapsilosis* and *Candida guilliermondii* (each n = 3, 6%), *Candida krusei* (n = 2, 4%), or *Candida* spp (unspecified) (n = 17, 34%) (not mutually exclusive; Table 3). The median time from admission to positive culture for *Candida* was 22 days (range, 1–112; n = 41; n = 9 patients were excluded from analysis [n = 2 patients admitted with known *Candida* infection, n = 1 patient with a culture result missing, and n = 6 patients with no positive culture for *Candida*]).

**Table 3.** Epidemiology of Infecting Fungal Organisms

Infecting Fungal Organism	Number of Patients	Percent (n = 50)
Patients with any fungal infection*	49^**†**^	98%
*Candida albicans*	17	34%
*Candida glabrata*	17	34%
*Candida guilliermondii*	3	6%
*Candida krusei*	2	4%
*Candida parapsilosis*	3	6%
*Candida* spp^‡^	17	34%
*Candida tropicalis*	1	2%
*Fusarium dimerum*	1	2%
*Saccharomyces cerevisiae*	2	4%
Yeast	2	4%

*Not mutually exclusive.

^**†**^One patient recorded as “probably liver abscess” at initiation had no positive cultures during the study period.

^‡^ *Candida* spp refers to situation in which infecting organism reported as *Candida* but species unspecified.

### Treatment and Outcomes

The median duration of treatment with anidulafungin was 15 days (IQR, 10.3–21.0); all patients received 200 mg on day 1 followed by 100 mg from day 2 onwards, as specified in the Summary of Product Characteristics [10]. During the observation period the manufacturer revised the formulation of anidulafungin injection, from an ethanol-based to a water-based product. Thirty-four patients (68%) received the ethanol-based product. Sixty-eight percent (34 of 50) completed their antifungal therapy on anidulafungin, and 32% (16 of 50) were switched to oral antifungal to complete therapy.

Of the 46 patients with no missing data, 35 (76%) had a favorable outcome (as defined above) and 7 (15%) required a change in or additional antifungal therapy. Nineteen patients (38%) in total died, 5 during and 14 after completing anidulafungin treatment. Causes of death were progressive liver disease and multiorgan failure in all but 1 case, which was attributable to multiple causes.

**Figure 1.**
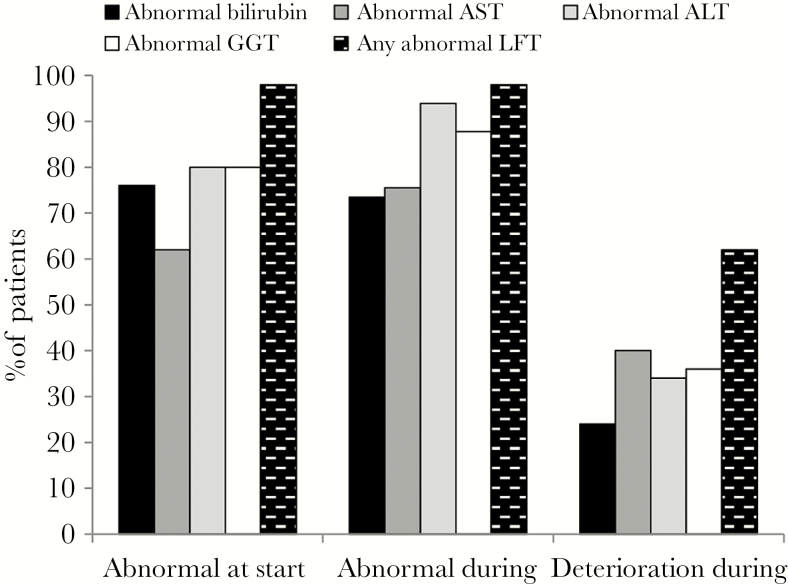
Liver function tests at initiation and during anidulafungin treatment. Deterioration: ≥2-fold increase in liver function test (LFT). ALT, alanine aminotransferase; ALP, alkaline phosphatase; AST, aspartate transaminase; GGT, gamma glutamyl transferase.

At the start of anidulafungin treatment, 49 of 50 (98%) patients had at least 1 abnormal LFT result and 48 of 50 (96%) patients had at least 1 abnormal LFT result during treatment. The proportion of patients with abnormal alkaline phosphatase or gamma glutamyl transferase was higher during treatment compared with the start of treatment (Figure 1). A deterioration in liver function was observed during anidulafungin treatment in 31 of 50 (62%) patients (deterioration in bilirubin, 12 of 50 [24%]; deterioration in aspartate transaminase, 20 of 50 [40%]; deterioration in alkaline phosphatase, 17 of 50 [34%]; deterioration in gamma glutamyl transferase, 18 of 50 [36%]) (Figure 1). At the cessation of anidulafungin treatment, 37 patients (74%) had abnormal LFTs including 25 patients (50%) with at least 1 LFT raised to more than twice the baseline level.

### Adverse Events

Serious adverse events were observed in 5 of 50 (10%) patients. Breakthrough *Candida* infections (positive culture obtained) were observed in 4 patients whilst receiving anidulafungin (2 *C glabrata*, 2 *C parapsilosis*), and 1 patient was observed to have *C glabrata* infection that persisted after more than 3 weeks of anidulafungin therapy. Of these patients, 3 were liver transplant patients (1 with autoimmune liver disease, 2 with alcoholic liver disease) and 2 had hepatopancreaticobiliary disease. All patients had multiple comorbidities and risk factors including the following: 3 patients had malignancy, 1 had diabetes, 4 had undergone major surgery, 3 had undergone dialysis, 2 had central lines, 3 had total parenteral nutrition, and 2 had concomitant viral infections. Sites of fungal infection included candidemia (2 patients), liver abscess and intra-abdominal infections (3 patients). These isolates were not tested for anidulafungin resistance because of nonavailability of a sensitive method in the laboratory.

## DISCUSSION

In this retrospective study, we report on a specialized group of patients with probable or proven *Candida* infection, who had liver disease and/or abnormal liver function and were admitted to a tertiary referral specialist unit. A significant proportion of patients suffered from multiorgan failure, and many had high illness severity with a wide range of comorbidities and high incidence of concomitant bacterial and viral infections. We believe that this is the reason for the high 38% mortality rate; none of these deaths were judged to be attributable to fungal infection or causally related to anidulafungin based on the clinical opinion of the study investigator. The severity of the patients’ chronic liver disease is evident in the fact that 13 patients had a MELD score of 30 or more, which predicts >50% mortality at 3 months [19]. In addition, all patients met at least 3 or more criteria for a diagnosis of SIRS, a serious condition related to systemic inflammation, organ dysfunction, and organ failure.

Despite the high illness severity, a favorable outcome was seen in more than three quarters (76.1%) of the 46 evaluable patients. This is similar to the primary outcome achieved in a previous European clinical trial in a general intensive care unit patient population (70.7% global response at end of intravenous therapy) [4]. The global response at the end of anidulafungin treatment in a comparative trial versus fluconazole [3] was also similar at 75.6%, even though this was a less severely ill cohort, and the microbiological criteria for response were less strict than in our study. An interesting observation in this study is the high incidence of non-albicans infections. We think this is due to a variety of reasons such as historical high usage of fluconazole as universal antifungal prophylaxis postliver transplantation (seen in the 70% of patients who had received fluconazole within the 30 days before starting anidulafungin) and the tertiary and international referral practice in our institution. Consistent with the patient population, 98% had abnormal LFTs at the start of anidulafungin and 96% had abnormal LFTs at 1 or more time points during treatment. Evaluation of the individual LFTs revealed more patients with abnormal alkaline phosphatase or gamma glutamyl transferase during treatment than at the start. In addition, a deterioration in liver function was observed in 62% of patients during treatment and 50% at cessation of treatment, not allowing to exclude a possible transient effect of anidulafungin on liver function. A similar phenomenon of transient increases in hepatic enzymes has been reported in clinical trials with anidulafungin, especially in patients with serious underlying disease and those treated with multiple concomitant medications [10], as was the case with our patient cohort. Liver function test results fluctuated in many patients at various points during and on cessation of anidulafungin; however, because of the ongoing abnormalities in liver function in this patient cohort due to the underlying liver disease, it is not possible to infer any causal relationship between anidulafungin and transient changes in liver function.

The main study limitation was that data collection was retrospective from both paper-based and electronic patient records. This meant that not all the data needed to determine outcomes defined in the study protocol were available for each patient; this limited our ability to fully assess clinical outcomes in individual study patients and resulted in a reduction in the sample size and, therefore, may have introduced a selection bias. The size of the cohort in this study limits the usefulness of performing subgroup analyses (in postliver transplant, chronic liver diseases, and hepatobiliary patients).

## CONCLUSIONS

In conclusion, in this group of patients with liver dysfunction and organ failure complicated by IFI, a favorable response was seen in more than 75% of patients treated with anidulafungin. Liver function tests worsened during anidulafungin treatment in many patients; however, this was usually reversible, because fewer patients had abnormal LFTs at cessation of treatment than at the start of treatment. We conclude that anidulafungin was efficacious and well tolerated by patients with decompensated liver disease or multiorgan failure and high-risk liver transplant recipients as treatment for proven or probable IFI. This study provides the largest body of real-world clinical evidence on anidulafungin to date in this highly specialized group of patients.
